# Retraction: Corrigendum: Upregulation of CCT-3 induces breast cancer cell proliferation through miR-223 competition and Wnt/b-catenin signaling pathway activation

**DOI:** 10.3389/fonc.2025.1575643

**Published:** 2025-03-04

**Authors:** Frontiers Editorial Office

The publisher retracts the 19 May 2022 article cited above.

Following publication, concerns were raised regarding the integrity of the images in the published figures. The authors failed to provide the raw data or a satisfactory explanation during the investigation, which was conducted in accordance with Frontiers’ policies. Given the concerns about the validity of the data, and the lack of raw data, the editors no longer have confidence in the findings presented in the article.

This retraction was approved by the Chief Editors of Frontiers in Oncology and the Chief Executive Editor of Frontiers. The authors received a communication regarding the retraction and had a chance to respond. This communication has been recorded by the publisher.

Frontiers would like to thank the users on PubPeer for bringing the published article to our attention.

